# Development and validation of a health belief model based instrument for measuring factors influencing exercise behaviors to prevent osteoporosis in pre-menopausal women (HOPE)

**DOI:** 10.1186/1471-2474-15-61

**Published:** 2014-03-01

**Authors:** Atoosa Soleymanian, Shamsaddin Niknami, Ebrahim Hajizadeh, Davoud Shojaeizadeh, Ali Montazeri

**Affiliations:** 1 Department of Health Education, Faculty of Medical Sciences, Tarbiat Modares University, Tehran, Iran; 2 Department of Biostatistics, Faculty of Medical Sciences, Tarbiat Modares University, Tehran, Iran; 3 Department of Health Education and Promotion, School of Public Health, Tehran University of Medical Science, Tehran, Iran; 4 Mental Health Research Group, Health Metrics Research Center, Iranian Institute for Health Sciences Research, ACECR, Tehran, Iran

## Abstract

**Background:**

The health belief model (HBM) is the most commonly used conceptual framework for evaluating osteoporosis health belief and behaviors. The purpose of this study was to develop and evaluate the psychometric properties of a health belief model based questionnaire for exercise behavior for preventing osteoporosis among women aged 30 years and over.

**Methods:**

This was a cross sectional study of a convenience sample of women aged 30 years and over in Tehran, Iran using a theory-based instrument (HOPE). The instrument contained 39 items covering issues relate to osteoporosis prevention behavior. In this methodological study, exploratory and confirmatory factor analyses were used for psychometric evaluation. The Cronbach’s alpha coefficient and Intraclass Correlation Coefficient (ICC) was used to evaluate the reliability of the scale.

**Results:**

In all 240 women participated in the study. The mean age of participant was 39.2 ± 7.8 years. The initial analysis extracted nine factors for the questionnaire that jointly accounted for 66.5% of variance observed. Confirmatory factor analysis showed that the data obtained was fit with Health Belief Model (HBM) and self-regulation construct (X^2^ = 1132.80, df = 629, P < 0.0001, CFI = 0.94, GFI = 0.91, RMSEA = 0.05 and SRMR = 0.06). The Cronbach’s alpha coefficient for the subscales ranged from 0.72 to 0.90 and Intraclass Correlation Coefficient (ICC) ranged from 0.71 to 0.98; well above acceptable thresholds.

**Conclusions:**

The HOPE was found to be appropriate instrument for measuring health belief and self-regulation for prevention of osteoporosis.

## Background

The World Health Organization (WHO) defined osteoporosis as ‘low bone mass and micro-architectural deterioration of bone tissue causes to increased bone fragility and consequent enhance in fracture [1]. Osteoporosis affects many millions worldwide and it has become a silent epidemic. It is termed the "silent thief" because there are often no symptoms until a fragility fracture occurs [2]. In Asian population, osteoporosis is more prevalent than the western countries because Asian population have lower body mass index and shorter height [3]. Furthermore, a lack of physical activity and low dietary calcium intake are common risk factors for osteoporosis in the Asian population [4].

A recent study from Iran reported that the prevalence of osteoporosis and osteopenia in at least one measured site among people aged 50 and over was 22.2% and 59.9% in women and 11.0% and 50.1% in men. Among individual younger than 50, 50.3% of women and 11.0% of men had reduced bone density [5,6]. However, there is evidence that increased bone mineral density due to positive lifestyle changes might reduce the incidence of osteoporosis [7]. The lifestyle changes include increased intake of dietary calcium and vitamin D and increased exercise [8].

The lack of physical activity is recognized as an important contributing factor to various health problems [9]. In a 2007 survey, approximately 15.0% of Iranian adults (4.7 million people) indicated that they do not have any physical activity and 40.0% of Iranian adults (31.6% of men and 48.6% of women) identified themselves as having low of physical activity category [10].

To make changes happen, understanding individual's health beliefs and attitudes to specific health issues are important. Reviews on health-related behavior has showed that individuals will generally not try to seek for diagnosis, prevention, or treatment for a condition unless they have minimal levels of related health motivation and information. Furthermore, these individuals must be potentially vulnerable, aware about the seriousness of their situation and convinced of the efficacy of health intervention [11]. Studies have demonstrated that individuals will be more likely to engage in the recommended health behaviors if they develop self-regulation abilities to change their health behaviors [12].

The main objective of this paper was to develop an instrument that can be used to explore factors influencing Iranian women's exercise behaviors for preventing osteoporosis. This study attempts to understand women’s health beliefs and the barriers that determine compliance with exercise to prevent osteoporosis among Iranian women. The framework for this study was built on the basis of health belief model and self-regulation construct of social cognitive theory.

## Methods

### Design and procedures

This was a cross-sectional study in order to develop a theory-based instrument for measuring factors influencing exercise behaviors among women aged 30 years and over. The participants were selected randomly from a population of females working in a ministry in Tehran, Iran. They were informed verbally about the aim of the study by the first author, and then asked if they agree to complete a self-administered questionnaire. Women were included in the study if they were aged 30 years and over, pre-menopausal, and having no history of osteoporosis. In all 250 women were approached. Of these 240 women met the inclusion criteria. Data were collected from January to December 2012.

### The instrument

A literature search was conducted to identify instruments that contain items related to factors influencing exercise behaviors. The search was guided by using combination of different keywords including exercise, physical activity, osteoporosis, HBM, social cognitive theory and self-regulation. The search engines included PubMed, Science Direct, and Google Scholar. Then, the following questionnaires were identified to create a new instrument based on health belief model and social cognitive theory. At last, the research team discussed about item selection and some items were excluded from each instrument because they either duplicated other items or failed to convey a clear expression of our intended objectives. The instruments and number of items are presented as follows:

1. The Osteoporosis Health Belief Scale (OHBS): The Osteoporosis Health Belief Scale is a well-known instrument for investigating beliefs associated with exercise and calcium intake. It consists of 39 items measuring 7 subscales: susceptibility, seriousness, benefits of exercise, benefits of calcium intake, barriers to exercise, barriers to calcium intake, and health motivation [13]. We selected 22 items from this questionnaire for measuring susceptibility (4 items), seriousness (4 items); benefits of exercise (5 items); barriers to exercise (5 items); and health motivation (4 items).

2. The Osteoporosis Self-Efficacy Scale (OSES): In order to measure confidence about osteoporosis preventing activities we used the OSES. It consists of 12 items relating to exercise (6 items) and nutrition (6 items). However we only used 5 items from the exercise section [13].

3. The Exercise Goal-Setting Scale (EGS): The scale contains 10 items relating to goal setting, self-monitoring, and problem solving. We used 8 items to measure self-regulation [14].

4. The Exercise Planning and Scheduling Scale (EPS): This is a 10-items questionnaire that measures scheduling and planning exercise as part of one’s daily routine. This scale also was used to measure self-regulation by selecting 4 items [14].

### Translation

The forward-backward-forward translation method was used to translate the 39-item questionnaire from English into Persian (the formal language of Iranians) as previously mentioned [15]. Three translators fluent in both English and Persian undertook the translation process. They were all experienced health care professionals who have been working for many years. In addition, in a period of pretesting (as a pilot study), the translated questionnaire was distributed to 30 employee women in order to test the degree of difficulty and clarity of questions, as well as the appropriateness and comprehensiveness of each item. Based on feedback from the pilot study the questionnaire was slightly modified before being used, and these were not included for any statistical procedures in the final study.

### Administration and scoring of the instrument

The final Persian version of the questionnaire was composed of 39 items and seven subscales (Susceptibility, Seriousness, Barriers, Benefits, Health motivation, Self-efficacy and Self-regulation). A five-point Likert format was used to measure each statement. Accordingly, the range of possible responses for each item was determined as follows: 1 = strongly disagree, 2 = disagree, 3 = neither agree nor disagree, 4 = agree and 5 = strongly agree.

### Sample

The sample size was estimated on the basis of planned procedure for performing factor analysis. Thus, as suggested to ensure a conceptually clear factor structure for analysis a sample of 5 to 10 women per item were thought [16]. The desired minimum required sample size was then determined to be 240.

### Statistical analysis

Descriptive statistics were used to explore the data and several procedures were applied to assess the psychometric properties of the questionnaire.

Validity: Construct validity was examined using exploratory factor analysis, confirmatory factor analysis and items-scale correlation. The exploratory factor analysis (principal component analysis) was applied to extract the factors. Kaiser-Mayer-Olkin (KMO) > 0.6 and Bartlett’s test for sphericity (P < 0.05) were considered for sampling adequacy for factor analysis. Any factor with an eigenvalue ≥ 1 was considered significant for factor extraction. The extracted factors were rotated orthogonally using varimax procedure. The acceptable level for factor loading of ≥ 0.40 was considered [17]. The confirmatory factor analysis was used for evaluating the coherence between the data and structure. The model fit was evaluated using multiple fit indices. The common indices chosen were: the chi-square statistics (χ^2^); normed chi-square (χ2/df); comparative fit index (CFI); goodness-of-fit index (GFI); and root mean square error of approximation (RMSEA). A good model fit is indicated by values of 0.90 or higher for the CFI, GFI and for the RMSEA, values of 0.05 or lower indicate a close fit and values less than 0.08 demonstrate an acceptable fit [18]. Finally, item-scale correlation was carried out to assess the extent to which an item was correlated to its hypothesized subscale. The Pearson correlation coefficient values equal or grater than 0.4 was considered satisfactory [19].

Reliability: The Cronbach's alpha coefficient was evaluated for internal consistency of the questionnaire [20]. A reliability coefficient of 0.70 or above was accepted as evidence of internal consistency for the instrument [21]. In addition, 40 women from the sample (n = 240) were randomly selected and agreed to complete the questionnaire twice with one to two week intervals in order to perform test–retest reliability analysis. intraclass correlation coefficients (ICC) was applied for each scale.

### Ethics

Ethics committee of Tarbiat Modares University approved the study. Participants gave their written consent and were assured about confidentiality and the right to withdraw at any time.

## Results

### The study sample

In all 240 employed women were entered into the study. The mean age of participants was 39.2 (SD = 7.8) years. Most women were married (74.2%) and had higher education (81.2%). The characteristics of the respondents are shown in Table 1.

**Table 1:** Demographic characteristics of the respondents (n = 240)

	**Number**	**%**
**Age**		
Mean (SD)	39.2 (7.8)	
**Marital status**		
Single	61	25.4
Married	179	74.6
**Education**		
Secondary	45	18.8
Higher	195	81.2
**Body mass index**		
< 18.5	2	0.8
18.5-24.9	126	52.5
25.0-29.9	78	32.5
≥ 30.0	34	14.2

### Exploratory factor analysis

Prior to evaluating the results of exploratory factor analysis (EFA) the Kaiser-Meyer-Olkin and Barlett’s tests were used. The value of KMO measurement was 0.81, (chi-square = 5.489, P < 0.0001), indicating that the sample size was adequate. Principal components analysis was carried out for all 39 items by using a varimax rotation. The primary analysis extracted nine factors that jointly accounted for 66.5% of variance observed. All items loaded under their respective theoretical constructs and each factor was labeled as follows: Factor 1: ‘Benefits’, Factor 2: ‘Self-efficacy’, Factors 3: ‘Susceptibility’, Factor 4: ‘Goal setting’, Factors 5, ‘Seriousness’, Factor 6: ‘Planning’, Factor7: ‘Barrier’, Factor 8: ‘Health Motivation’ and Factor 9: ‘Monitoring’. Table 2 presents a summary of the 39 items, factors and factor loadings. In the next step we excluded those factors that implied the self-regulation concept (Factor 4, Factor 6, and Factor 9) and re-analyzed the data with similar method. The results obtained from the analysis indicated that the six factors jointly accounted for the 63.2% of variance observed.

**Table 2:** Factor loading for the HOPE obtained from the exploratory factor analysis*

**Items (number)**	**Factor 1**	**Factor 2**	**Factor 3**	**Factor 4**	**Factor 5**	**Factor 6**	**Factor 7**	**Factor 8**	**Factor 9**
I often set exercise goals (1)	.136	.089	-.003	**.785**	.094	.039	.080	-.051	.056
I usually have more than one major exercise goal (2)	.087	.104	-.075	**.787**	.072	.163	.053	.101	.092
2. My exercise goals help to increase my motivation for doing exercise (3)	.190	.019	.102	**.780**	.086	.053	.105	.095	.147
I tend to break more difficult exercise goals down into a series of smaller goals (4)	.112	.076	-.036	**.680**	-.033	.247	-.097	.057	.124
I usually keep tract of my progress in meeting my goals (5)	.216	.002	.063	.506	-.046	.251	.004	.033	**.565**
I have developed a series of steps for reaching my exercise goals (6)	.123	.080	.014	.356	-.030	.058	-.002	.142	**.673**
I usually achieve the exercise goals I set for myself (7)	.044	.143	-.092	.020	.123	.123	-.103	.003	**.489**
If I do not reach an exercise goal, I analyze what went wrong (8)	.103	.043	-.035	.164	.148	.354	.076	.086	**.652**
I schedule all events in my life around my exercise routine (9)	.081	.271	.000	.145	-.030	**.681**	.041	.124	.219
I schedule my exercise at specific times each week (10)	.038	.187	.010	.294	.100	**.752**	.030	.076	.062
I plan my weekly exercise schedule (11)	-.040	.139	-.024	.255	.101	**.798**	.055	.157	.059
I write my planned activity sessions in an appointment book or calendar (12)	-.047	.125	-.018	-.040	-.058	**.642**	.068	.053	.318
my chances of getting osteoporosis are high (13)	.090	.079	**.894**	.039	.142	-.118	-.114	.005	.000
Because of my body build, I am more likely to develop osteoporosis (14)	.069	-.022	**.845**	.001	.230	.043	-.075	.044	.060
My family history makes it more likely that I will get osteoporosis (15)	.059	.039	**.905**	.000	.113	-.123	-.054	-.008	-.056
It is extremely likely that I will get osteoporosis (16)	.069	-.035	**.647**	-.039	.186	.153	-.125	.041	-.110
If I had osteoporosis I would be crippled (17)	.085	-.036	.250	.050	**.679**	.145	-.177	.077	.027
It would be very costly if I got osteoporosis (18)	.256	.049	.151	.056	**.874**	-.074	-.079	.007	.076
When I think about osteoporosis I get depressed (19)	.268	.042	.146	.059	**.852**	-.073	-.090	-.010	.086
It would be very serious if I got osteoporosis (20)	-.046	-.040	.213	.066	**.724**	.111	-.194	.109	.033
Regular exercise prevents problems that would happen from osteoporosis (21)	**.785**	.171	.117	.086	.169	-.013	-.055	.147	.048
Regular exercise helps to build strong (22)	**.892**	.178	.054	.125	.107	.023	-.044	.046	.067
Exercising to prevent osteoporosis also improves the way my body looks (23)	**.815**	.216	.016	.192	.158	-.048	-.070	.188	.050
Regular exercise cuts down the chances of broken bones (24)	**.838**	.181	.032	.180	.081	.014	-.067	.049	.038
I feel good about myself when I exercise to prevent osteoporosis (25)	**.615**	.224	.171	.091	.056	.053	.015	.198	.270
I feel like I am not strong enough to exercise regularly (26)	-.027	.068	-.228	.098	-.137	.165	**.584**	.008	-.186
I have no place where I can exercise (27)	-.006	-.106	-.106	-.002	-.177	.199	**.671**	-.145	-.041
My spouse or family discourages me from exercising (28)	.038	-.189	.024	.001	-.155	-.023	**.603**	-.016	-.144
Exercising regularly makes me uncomfortable (29)	-.054	.057	-.163	-.006	-.014	.007	**.755**	.003	.165
Exercising regularly upsets my every day routine (30)	-.176	.179	.030	.084	-.045	-.115	**.777**	.055	.100
I can begin a new or different exercise program (31)	.184	**.800**	-.021	.019	-.034	.050	-.077	.013	.066
I can change my exercise habit (32)	.257	**.748**	.078	.087	.058	.109	-.138	.087	-.004
I do exercise even if they are difficult (33)	.184	**.802**	.024	.049	.025	.196	.135	.094	.092
I do exercise at least 30 minutes each day (34)	.087	**.670**	-.034	.081	.012	.150	-.011	.134	.164
I do the type of exercises that I am supposed to do (35)	.177	**.690**	.023	.103	-.042	.177	.109	.229	.013
I look for new information related to health (36)	.157	.063	.068	.051	.042	.100	.008	**.799**	.083
Keeping healthy is very important for me (37)	.425	.099	-.033	.124	.031	.144	-.043	**.623**	-.092
I try to discover health problems early (38)	.156	.267	.062	.084	.133	.028	-.062	**.751**	.059
I have a regular check-up even when I am not sick (39)	-.037	.209	-.055	-.031	-.035	.305	-.022	**.524**	.332
Eigenvalues	8.259	4.842	2.820	2.430	2.104	1.608	1.522	1.212	1.148
% of variance	21.177	12.416	7.231	6.230	5.396	4.123	3.904	3.108	2.942

*Factor loading equal or greater than 0.4 was considered acceptable.

Factor 1: Benefit, Factor 2: Self-efficacy, Factor 3: Susceptibility, Factor 4: Goal setting, Factor 5: Seriousness, Factor 6: Planning, Factor 7: Barrier, Factor 8: Health motivation, Factor 9: Monitoring.

### Confirmatory factor analysis

**Figure 1:**
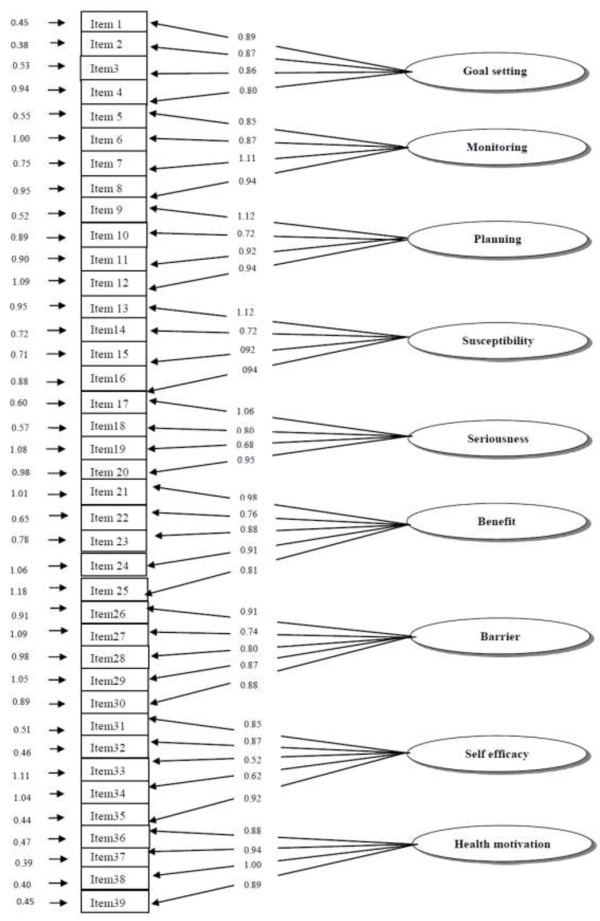
Factor loading for the HOPE.

Confirmatory factor analysis was used for testing the construct validity of the nine-factor model extracted from the EFA (Figure 1). The fit indices for the 39 items-model were X^2^ = 1132.80, df = 629, P < 0.0001, CFI = 0.94, GFI = 0.91, RMSEA = 0.05 and SRMR = 0.06 indicating a good fit to the data.

### Item-scale correlation

Item-scale correlation is presented in Table 3. As shown all items were correlated with its own hypothesized subscale, lending further support for the construct validity of the instrument.

**Table 3:** Subscales and item-scale correlation for the HOPE

**Items**	**Item mean**	**SD**	**Item-scale correlation**
**Self-regulation (Mean =44.10 SD = 7.43 Range 12–60)**			
1	4.17	0.88	0.551
2	4.13	0.91	0.651
3	4.33	0.76	0.612
4	3.93	0.91	0.627
5	3.93	0.87	0.725
6	3.94	0.82	0.605
7	3.53	0.98	0.680
8	3.46	0.98	0.669
9	3.53	1.05	0.678
10	3.36	1.13	0.720
11	3.16	1.11	0.713
12	2.60	0.99	0.537
**Susceptibility (Mean =12.26 SD = 3.72 Range 4–20)**			
13	3.19	1.04	0.910
14	3.23	1.07	0.877
15	2.97	1.08	0.891
16	2.86	1.15	0.742
**Seriousness (Mean =13.92 SD = 3.62 Range 4–20)**			
17	2.99	1.18	0.801
18	3.72	1.00	0.887
19	3.90	1.00	0.869
20	3.30	1.13	0.799
**Benefit (Mean =21.56 SD = 3.19 Range 5–25)**			
21	4.25	0.77	0.849
22	4.33	0.69	0.912
23	4.45	0.65	0.891
24	4.40	0.73	0.871
25	4.12	0.86	0.774
**Barrier (Mean =15.81 SD = 4.13 Range 5–25)**			
26	3.20	1.20	0.689
27	2.95	1.20	0.732
28	3.55	1.17	0.638
29	2.92	1.17	0.740
30	3.16	1.10	0.732
**Self-Efficacy (Mean =18.66 SD = 3.69 Range 5–25)**			
31	3.71	0.91	0.797
32	3.81	0.83	0.788
33	3.78	0.91	0.856
34	3.60	0.98	0.757
35	3.74	0.98	0.792
**Health motivation (Mean =15.65 SD = 2.76 Range 4–20)**			
36	4.14	0.78	0.750
37	4.52	0.70	0.671
38	3.92	1.01	0.813
39	3.06	1.17	0.741

### Instrument reliability

The Cronbach's alpha coefficient applied separately for each factor. The Cronbach's alpha coefficient for factors was found to be between 0.72 and 0.90, indicating an acceptable internal consistency for the instrument. No item was predicted to significantly increase the scale reliability if omitted. Thus no omission was made at this stage.

To test stability, test-retest analysis was performed. The results showed satisfactory statistics. Intraclass correlation coefficients (ICC) for each factors ranged between 0.71 and 0.98. Table 4 shows a summary of the Cronbach's alpha coefficient and ICC values for the instrument.

**Table 4:** Measures of internal consistency and stability

	**Number of items**	**Cronbach’s alpha**	**ICC**
**Self-regulation**	12	0.862	0.754
**Susceptibility**	4	0.807	0.776
**Seriousness**	4	0.781	0.976
**Benefits physical activity**	5	0.901	0.743
**Barriers physical activity**	5	0.727	0.812
**Self-Efficacy**	5	0.856	0.876
**Health motivation**	4	0.868	0.760

## Discussion

This study evaluated a newly developed instrument for assessing exercise behaviors in order to prevent osteoporosis. The instrument was theory driven and was developed using several items from existing questionnaires to ensure that this new instrument covers all theoretical concepts for adopting a healthy behavior. The tool was included susceptibility, seriousness, barriers, benefits, self-efficacy, health motivation and self-regulation subscales all reflecting on one’s motivation, ability and behavior to perform exercise for preventing osteoporosis.

Psychometric studies should be performed to standardize a scale and confirm that it is able to produce appropriate information. The novel contribution of the current study relies on the fact that we added a self-regulation construct to the health belief model. As such the exploratory factor analysis indicated that the model explained 66.5% of variances observed which is well above previous studies assessing the model without self-regulation construct [22,23]. In addition the confirmatory factor analysis indicated an improved fit indices for the current model provided by this study where all indices almost showed perfect results.

It is well known that the health belief model is based on value expectancy theory of behavior. This latter theory proposes that, in general, a behavior depends on how much an individual values a certain goal and on an individual's judgment that a particular action will attain that goal [24]. If the goal is to avoid a health problem, the individual must feel personally vulnerable to the problem (perceived susceptibility), judge that the problem potentially is serious (perceived severity), believe that particular action can be beneficial in decreasing the health threat (perceived benefit), and will not face obstacle in performing that particular action (perceived barriers) [25]. In addition the ability that one can successfully perform a behavior, requires confidence (self efficacy) [26]. The HBM has been used in a wide range of health behaviors investigations including studies on prediction exercise behavior. For example Gristwood showed that individual values and beliefs had a noticeable impact on physical activity engagement and were significant predictors of current and future health behaviors in older adults [27]. Gould & Weinberg identified when this model used for physical activity engagement of older adults, it explained the likelihood of an individual engaging in physical activity due to the perceived threats and the potential benefits could far outweigh the risks [28]. Also several studies have shown that the HBM constructs were significant in predicting osteoporosis prevention behaviors such as physical activity [29,30]. However, changing behaviors especially behaviors such as exercise needs long-term commitments. Thus ability to schedule and plan to adopt a behavior requires a sense of choice of fullness (self-regulatory). In other words, adopting such behaviors are unlikely to be performed out of habit or automatically without any mindful decision [31]. In fact self-regulation largely conveys a concept that includes goal setting, goal striving, and dealing with a series of challenges that individuals may face when trying to attain something that is important [32]. Self-regulatory behaviors are therefore planned, reflective and consciously directed rather than automatic, non-conscious and spontaneous actions [33]. This is why it is argued that health interventions must modify individual's health beliefs, increase self-efficacy, and reflect personal goals.

This newly developed scale may be especially useful and helpful to health professionals. Health professionals will be able to assess the health beliefs and self-regulation of individuals for adopting health behaviors. Indeed it seems that the future research should focus on how differently or similar the items may be interpreted or the questionnaire perform in other ethnic populations/countries.

### Limitations

Although this study was a theory based and introduced a newly developed instrument for assessing factors influencing exercise behaviors in order to prevent osteoporosis behavior, it had some limitations that warrant further investigation. For instance we know that increased intake of dietary calcium and vitamin D also contribute to preventing osteoporosis while we only focused on regular exercise. In addition the study participants were well educated pre-menopausal employed women aged 30 years and over. Therefore, the findings might not be generalized to all Iranian women. Furthermore one should note that the study did not look a predictive validity or attempt to correlate the questionnaire with any measure of actual exercise of physical activity behavior.

## Conclusions

The results indicated that the HOPE is a reliable and valid instrument for measuring factors influencing exercise behavior in order to prevent osteoporosis among women. In addition the finding suggest that adding self-regulation to health belief model might improve the model to a good extent.

## Competing interests

Authors declare that they have no competing interests.

## Authors’ contribution

AS was the main investigator, designed the study, collected the data and wrote the first draft. SN was the study supervisor. EH contributed to the statistics. DS was the study consultant. AM critically reviewed the manuscript, responded to the reviewers comments and provided the final draft. All authors read and approved the final manuscript.

## Pre-publication history

The pre-publication history for this paper can be accessed here:

http://www.biomedcentral.com/1471-2474/15/61/prepub
